# Overcoming heterogeneity and immunosuppression: novel strategies in adoptive therapy for biliary tract cancer

**DOI:** 10.3389/fimmu.2026.1771318

**Published:** 2026-02-25

**Authors:** Yifan Zhu, Ge Xiong, Mingcheng Guan, Di Sun, Yanchao Guo, Jun Peng, Hong Zhu

**Affiliations:** 1 Department of Medical Oncology, The First Affiliated Hospital of Soochow University, Suzhou, Jiangsu, China; 2 Department of Medical Oncology, Affiliated Hospital of Jiangnan University, Wuxi, Jiangsu, China

**Keywords:** adoptive cell immunotherapy, biliary tract cancer, chimeric antigen receptor T-cell therapy, cytokine-induced killer cells, natural killer cells, T-cell receptor-engineered T cells, tumor-infiltrating lymphocytes

## Abstract

**Background:**

Biliary tract cancer (BTC) is a highly heterogeneous malignancy originating from the biliary epithelium or gallbladder mucosa, characterized by strong invasiveness and poor prognosis. Although surgery remains the primary curative strategy, most patients are diagnosed at advanced stages, limiting surgical opportunities. The traditional gemcitabine plus cisplatin chemotherapy regimen, although a standard treatment, has limited efficacy and often leads to drug resistance. In recent years, adoptive cell immunotherapy has emerged as a promising new avenue for BTC treatment.

**Main body:**

This review systematically elaborates on the research progress of various ACT strategies in BTC, including chimeric antigen receptor T cells, tumor-infiltrating lymphocytes, natural killer cells, cytokine-induced killer cells, and T-cell receptor-engineered T cells. Furthermore, it comprehensively analyzes current key challenges and discusses future directions and optimization strategies regarding these therapies.

**Conclusion:**

This review summarizes recent progress in adoptive cell therapy for biliary tract cancer and discusses optimization strategies to facilitate clinical translation.

## 1 Introduction

Biliary tract cancer (BTC) is primarily classified into three clinical subtypes based on anatomical location: intrahepatic cholangiocarcinoma (iCCA), extrahepatic cholangiocarcinoma (eCCA), and gallbladder cancer (GBC). Extrahepatic cholangiocarcinoma can be further subdivided into perihilar and distal types, with significant differences in clinical presentation, treatment strategies, and prognosis among subtypes. The causes of biliary tract cancer are complex and varied. Established risk factors include gallstones, primary sclerosing cholangitis, hepatolithiasis, liver fluke infection, chronic viral hepatitis, and cirrhosis. In recent years, metabolic factors such as obesity and non-alcoholic steatohepatitis have been increasingly linked to a higher risk of BTC (1, 2). The global incidence of BTC is rising, particularly for the iCCA subtype. Epidemiological data show that between 1990 and 2019, the number of BTC cases and related deaths increased by 84.8% and 81.8%, respectively. The prognosis for BTC is very poor, with a median overall survival of approximately 9 months and a one-year survival rate of about 51% (3, 4). BTC also shows high molecular and genomic heterogeneity. This heterogeneity not only drives cancer progression but also poses major challenges for precision therapy. Due to its insidious onset, rapid progression, limited treatment options, and dismal prognosis, BTC constitutes a major social and healthcare burden (5).

Surgery is the first-choice curative treatment for BTC. However, the often asymptomatic nature of early-stage disease results in the majority of patients being diagnosed at an advanced stage. This substantially limits the applicability of surgical intervention and locoregional therapies, with only approximately 22% of patients ultimately qualifying for resection (3, 6). For patients with unresectable advanced disease, systemic pharmacotherapy is the mainstay. The combination of gemcitabine and cisplatin is established as the standard first-line chemotherapy regimen for BTC (7). However, traditional chemotherapy offers limited benefits. It provides only short-term remission for some patients, with most tumors rapidly developing resistance (8). While targeted therapies based on molecular profiling have emerged as an alternative to overcome chemotherapy resistance, their clinical application is constrained by challenges including limited biomarker detection rates, narrow applicability, and acquired resistance.

In recent years, with in-depth exploration of the tumor immune microenvironment and immunology, immunotherapy has gradually become a major research focus in cancer treatment (9, 10). This strategy enhances anti-tumor immune responses and overcomes immune evasion, demonstrating strong therapeutic potential. Immune checkpoint inhibitors (ICIs), particularly antibodies targeting programmed cell death protein 1 (PD-1) and programmed cell death ligand 1 (PD-L1), are now part of standard first-line regimens for many cancers (11). For BTC, regimens such as cisplatin plus gemcitabine with durvalumab or cisplatin plus gemcitabine with pembrolizumab are recommended as first-line options (12, 13). However, ICI monotherapy shows limited efficacy in BTC, with the majority of patients developing treatment resistance (14). This treatment resistance is closely linked to the immunosuppressive tumor microenvironment (TME) in BTC. The TME consists of tumor cells, immune cells, and stromal cells, and it is the key factor that limits ICI effectiveness and causes resistance (15).

Adoptive cell therapy (ACT) is an emerging tumor immunotherapy strategy centered on utilizing immune effector cells to attack tumors. The standard procedure can be summarized in three key steps: first, collecting immune-reactive cells from the patient’s peripheral blood mononuclear cells or tumor tissue; second, activating and expanding these cells ex vivo; and finally, reinfusing the expanded effector cells into the patient to mediate antitumor immunity (16). ACT has proven highly effective in treating hematological malignancies, primarily based on efficient targeting of specific antigens like cluster of differentiation (CD) 19 and B-cell maturation antigen. Its application is continuously expanding into the realm of solid tumors, where growing preclinical and clinical data are confirming its potential and paving the way for broader use (17–21).

**Figure 1:**
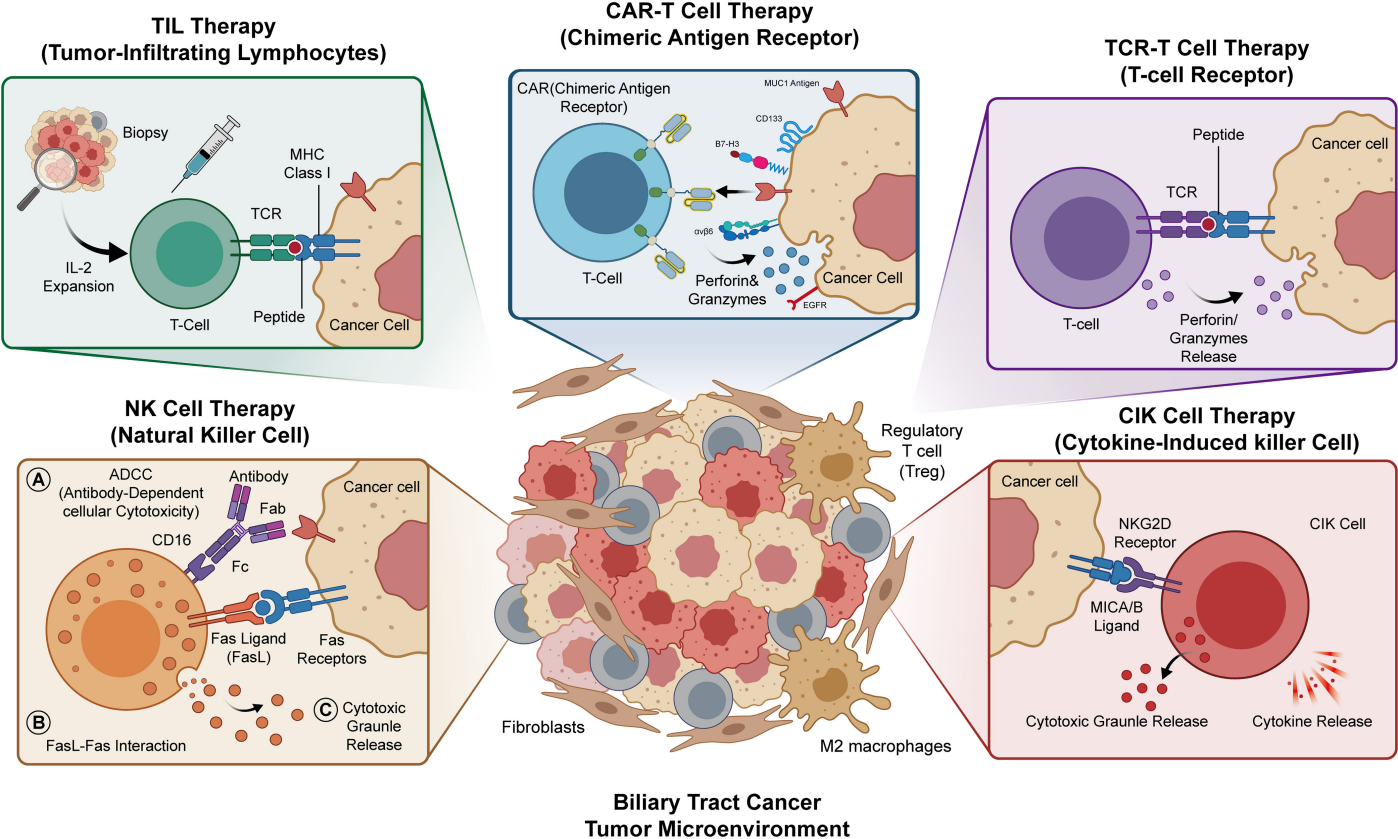
Diverse strategies of adoptive cell immunotherapy for the treatment of biliary tract cancer. The central panel illustrates the complex tumor microenvironment of biliary tract cancer. The surrounding panels depict the mechanisms of action for five major cell-based immunotherapies: CAR-T, TIL, NK, CIK and TCR-T therapies.

BTC, as a malignancy characterized by both high heterogeneity and strong immunosuppressive properties, has limited responses to conventional therapies, creating an urgent clinical need for new strategies. In this context, various adoptive cell therapy modalities, such as chimeric antigen receptor T cells (CAR-T), tumor-infiltrating lymphocytes (TIL), cytokine-induced killer cells (CIK), natural killer cells (NK), and T-cell receptor-engineered T (TCR-T) cells, are being extensively explored for BTC treatment (**Figure 1**). This review will detail the research progress of these ACT strategies in BTC through dedicated sections. We will also discuss the key challenges currently faced in the field and share perspectives on potential future directions.

## 2 Chimeric antigen receptor T-cell therapy

Chimeric antigen receptor T-cell therapy is an immunotherapy strategy that involves genetically engineering a patient’s own T cells to express chimeric antigen receptors targeting specific tumor antigens. It has recently emerged as a promising direction in BTC treatment. The structure of CARs has evolved through generations, but its core consistently comprises three functional domains: an extracellular antigen recognition domain, a transmembrane domain, and an intracellular signaling domain (22). The extracellular domain is typically a single-chain variable fragment (scFv) derived from a monoclonal antibody. This fragment is generated by linking the variable regions of the antibody heavy and light chains with a flexible linker. It is responsible for the specific recognition of epitopes on tumor cell surfaces, thereby forming the molecular basis of CAR targeting. The transmembrane domain, serving as an anchor connecting the extracellular and intracellular parts, is usually derived from molecules like CD3ζ, CD8, or CD28. This domain not only anchors the CAR to the T-cell membrane but also plays a crucial role in signal transduction and receptor stability. The intracellular signaling domain is the core component that activates T-cell functions. The first-generation CARs only contained the primary CD3ζ signaling domain, which often resulted in limited T-cell activation, poor expansion, and insufficient persistence. To overcome these limitations, a key innovation in second-generation CARs was the addition of specific co-stimulatory domains like CD28 or 4-1BB, fused directly to the CD3ζ chain. This pivotal enhancement significantly improved T-cell activation, proliferation, cytokine production, and persistence. Building on this, third-generation CARs further incorporated two distinct co-stimulatory domains, aiming to synergistically amplify anti-tumor responses. However, challenges posed by the solid TME prompted the development of fourth-generation CARs, also known as T cells redirected for universal cytokine killing (TRUCKs). These are engineered based on earlier generations but are equipped with inducible cytokine modules. Upon recognizing tumor antigens, these CAR-T cells trigger direct cytotoxicity alongside localized cytokine release. This helps remodel the immune microenvironment, recruit and activate innate immune cells, and ultimately achieve more potent tumor eradication (23, 24). Currently, fifth-generation CAR-T therapies are still under development and refinement. Their core features include a modular design philosophy employing a “universal receptor + targeting module” split-receptor system; the use of gene editing technologies like clustered regularly interspaced short palindromic repeats/CRISPR-associated protein 9 (CRISPR/Cas9) or transcription activator-like effector nuclease for precise cell engineering; and the integration of T-cell receptor signaling, co-stimulatory signals, and cytokine signaling pathways for synergistic CAR-T cell activation. Such designs aim to improve the universality, persistence, resistance to exhaustion, and safety of CAR-T cells, opening new potential strategies for solid tumor immunotherapy (25–27). In BTC, CAR-T therapy primarily targets tumor-associated antigens such as Mucin 1 (MUC1), Epidermal Growth Factor Receptor (EGFR), integrin alpha-v beta-6 (αvβ6), CD133, and B7 Homolog 3 (B7-H3) (**Table 1**).

**Table 1:** Key target antigens in CAR-T cell therapy for biliary tract cancer.

Target antigen	Key therapeutic strategy & mechanism	Preclinical/Clinical outcomes	References
MUC1	• 2nd generation CAR-T targeting MUC1 • 4th generation CAR-T targeting MUC1 • PD-1–CD28 switch receptor to overcome PD-L1 inhibition	• Significant lysis of MUC1-high cells and disruption of 3D spheroids. • Dual-function CAR-T showed enhanced proliferation and reduced exhaustion in high PD-L1 conditions.	(31, 32, 35)
EGFR	• Autologous CAR-T-EGFR infusion following conditioning chemotherapy	• Phase I Trial: 1 Complete Response, 10 Stable Disease, median PFS of 4 months. • Toxicities included manageable mucosal/cutaneous reactions and fever.	(38, 39)
Integrin αvβ6	• 2nd and 4th generation CAR-T • 5th generation CAR-T secreting anti-PD-L1 scFv	• 4th generation showed superior persistence over 2nd generation. • 5th generation successfully blocked PD-1/PD-L1 pathway, showing deeper infiltration in 3D models.	(43, 44)
CD133	• 4th generation CAR-T targeting CD133 • Sequential “Cocktail” therapy (following EGFR CAR-T)	• Strong cytotoxicity and cytokine release (IFN-γ, TNF-α) *in vitro*. • Case report: Patient achieved PR for 4.5 months after sequential treatment.	(39, 48)
B7-H3	• B7-H3-specific CAR-T • Combination with TBK1 inhibition	• Significant inhibition of tumor progression in xenografts. • TBK1 inhibition sensitized tumors to cytokines and prevented CAR-T exhaustion.	(52, 53)

### 2.1 MUC1

Mucin 1 is a key type I transmembrane glycoprotein responsible for maintaining epithelial barrier function under normal physiological conditions. In BTC and other epithelial cancers, MUC1 expression becomes dysregulated. It is overexpressed, exhibits aberrant glycosylation, and loses its apical membrane polarity. These alterations collectively promote malignancy by enhancing tumor proliferation, invasion, and metastasis (28, 29). Studies have shown that MUC1 expression is significantly increased in cholangiocarcinoma. Widespread expression (over 50%) tends to correlate significantly with the presence of metastasis. It is also closely associated with poor prognostic features such as local invasion (30). These findings suggest that MUC1 plays a key role in driving the aggressive progression of cholangiocarcinoma (CCA). Additionally, research confirms that second-generation anti-MUC1 CAR-T cells can lyse Tn-MUC1-positive CCA cells. In xenograft models, these CAR-T cells markedly suppressed tumor growth and induced extensive infiltration of CD3+ T-cells (31). Supimon et al. further advanced the field. The team successfully engineered fourth-generation anti-MUC1 CAR-T cells incorporating CD28, CD137, and CD27 signaling domains. These cells demonstrated significant cytotoxicity against MUC1-high cells (66.03% specific lysis) and effectively disrupted 3D tumor spheroids, whereas cytotoxicity against immortalized normal cholangiocytes was negligible, further supporting safety (32). Some studies suggest the overexpression of MUC1 frequently correlates with elevated levels of programmed death-ligand 1 (PD-L1). The binding of PD-L1 to PD-1 on CAR-T cells triggers an inhibitory signal, leading to T-cell exhaustion (33, 34). To address this, Supimon et al. created anti-MUC1 CAR-T cells that also express a PD-1–CD28 switch receptor. This receptor combines the extracellular part of PD-1 with the intracellular domain of CD28, turning an inhibitory signal into a stimulatory one. Experiments showed that the dual-function CAR-T cells proliferated and killed better in high PD-L1 conditions. They also maintained anti-tumor activity in long-term co-cultures and 3D models, with fewer exhaustion markers (35). Although no MUC1-targeted CAR-T product is yet approved for BTC treatment, relevant early clinical trials (e.g., NCT03633773) are exploring its safety and feasibility in BTC.

### 2.2 EGFR

Epidermal Growth Factor Receptor is overexpressed in various epithelial tumors, including BTC, playing a key role in tumor cell proliferation, differentiation, migration, and survival, making it a potential immunotherapeutic target (36, 37). In a phase I clinical trial, patients with advanced unresectable or metastatic EGFR-positive (>50% expression) BTC received CAR-T-EGFR cell therapy. Patients underwent preconditioning chemotherapy with nab-paclitaxel and cyclophosphamide before infusion of autologous CAR-T-EGFR cells. Results showed that among evaluable patients, 1 achieved complete response, and 10 had stable disease, with a median progression-free survival (PFS) of 4 months (range: 2.5–22 months). Regarding safety, while the infusion was generally tolerated, grade ≥3 fever/chills were observed. Notably, on-target/off-tumor toxicities manifested as grade 1–2 mucosal and cutaneous reactions due to basal EGFR expression in normal tissues. One patient experienced acute pulmonary edema associated with cytokine release, which was reversible with tocilizumab (38). A case report further validated the potential of CAR-T-EGFR in advanced CCA. A patient with advanced CCA achieved an 8.5-month partial response after CAR-T-EGFR treatment but developed grade 2 lichen striatus-like skin rash. Subsequent sequential CAR-T-CD133 treatment resulted in a sustained response, providing evidence for multi-target strategies to overcome heterogeneity while highlighting the need to monitor epithelial damages (39).

### 2.3 Integrin αvβ6

Integrin αvβ6 is a receptor protein highly expressed in epithelial-derived tumors but with very low expression in normal tissues. It not only participates in tumor invasion and metastasis but may also influence treatment efficacy by modulating the immunosuppressive TME. These characteristics make it an ideal target for solid tumor immunotherapy (40, 41). In CCA, the expression rate of integrin αvβ6 is as high as 73.3%, and its high expression is significantly associated with poor patient prognosis (42). Based on this, Phanthaphol et al. developed second-generation (A20-2G CAR) and fourth-generation (A20-4G CAR) CAR-T cells targeting integrin αvβ6. *In vitro* experiments demonstrated that both generations of CAR-T cells exhibited antigen-specific cytotoxicity against αvβ6-positive CCA cell lines, with negligible activity against antigen-negative controls. This activity was further confirmed in 3D tumor spheroid models, where the cells showed strong infiltration and killing efficacy. Furthermore, the study indicated that fourth-generation CAR-T cells outperformed second-generation CAR-T cells in terms of proliferative capacity and long-term anti-tumor activity (43). To further overcome PD-L1-mediated immunosuppression in CCA, the research team constructed fifth-generation CAR-T cells (A20 CAR5). These cells target integrin αvβ6 and simultaneously secrete an anti-PD-L1 scFv which locally blocks the PD-1/PD-L1 pathway. In long-term co-culture and repeated tumor challenge experiments, A20 CAR5 T cells demonstrated stronger persistence and cytotoxicity. These cells also showed deeper tumor infiltration in 3D spheroid models, further validating their potential to counteract the immunosuppressive TME (44). In summary, integrin αvβ6 is a therapeutic target with significant clinical value in BTC. Targeting it exerts dual antitumor effects by directly killing tumor cells and potentially modulating the immunosuppressive TME via anti-fibrotic activity. This provides a crucial mechanism to overcome a major obstacle in current cell immunotherapy.

### 2.4 CD133

CD133 is a five-transmembrane glycoprotein widely recognized as a marker for cancer stem cells in various solid tumors. This protein plays a key role in tumorigenesis, metastasis, drug resistance, and recurrence (45). In biliary tract cancers, elevated CD133 expression is significantly associated with poor prognosis and high recurrence rates. Approximately 67.6% of cholangiocarcinoma tissue samples show high CD133 expression (46, 47). Fourth-generation CAR-T cells targeting CD133 demonstrated strong cytotoxicity *in vitro*, with a maximum specific lysis of 57.59% ± 9.62%, accompanied by significant upregulation of IFN-γ and TNF-α (48). Clinically, Feng et al. reported a case of an advanced CCA patient. After developing resistance to CAR-T-EGFR therapy, this patient received CD133-targeted CAR-T therapy. The patient achieved a partial response (PR) that lasted 4.5 months (39). This case first demonstrated the feasibility of a “CAR-T cocktail therapy” (sequential targeting of different antigens) in solid tumors.

### 2.5 B7-H3

B7-H3 is a member of the B7 immunoregulatory protein family and is overexpressed in various solid tumors. This high expression is closely linked to an immunosuppressive microenvironment and poor clinical prognosis. Recently, B7-H3 has become an important target for CAR-T therapy in solid tumors. It shows particular promise for treating refractory cancers like BTC (49–51). Studies confirm that B7-H3 is commonly highly expressed in patient-derived organotypic tumor spheroids and CCA cell lines, and the degree of expression correlates positively with the killing efficiency of B7-H3-specific CAR-T cells (52). Preclinical experiments further indicate that these CAR-T cells can effectively eliminate B7-H3-positive CCA cells *in vitro* and significantly inhibit tumor progression while prolonging survival in xenograft mouse models (53). Although B7-H3.CAR-T shows significant anti-tumor activity in preclinical models, its efficacy in solid tumors remains limited by TME-induced T-cell dysfunction. To overcome this, researchers explored combining it with TANK-binding kinase 1 (TBK1) inhibition to enhance efficacy. TBK1 inhibition not only sensitizes tumor cells to IFNγ and TNFα released by CAR-T cells but also prevents CAR-T cell exhaustion and promotes their proliferation and effector function, thereby significantly improving treatment outcomes in PDOTS and monoculture tumor spheroid models (52). These studies establish B7-H3 as a promising target for CAR-T therapy in BTC.

## 3 Tumor-infiltrating lymphocyte therapy

Tumor-infiltrating lymphocyte therapy is an adoptive cell treatment based on T lymphocytes isolated from the patient’s own tumor tissue. TIL therapy relies on isolating T cells with inherent tumor recognition capability from the TME, expanding them ex vivo, and reinfusing them into the patient to enhance anti-tumor immune responses. Compared to most other adoptive cell therapies, TIL therapy does not require genetic engineering. This may lower the risk of adverse events associated with genetic modification. Simultaneously, it mediates specific tumor cell killing, demonstrating good targeting and therapeutic potential (54, 55). It has shown significant clinical efficacy in solid tumors like melanoma and has recently expanded into research for gastrointestinal malignancies (56–58).

In BTC, TILs demonstrate the potential to target tumor-specific antigens. Evidence shows that TILs can recognize neoantigens derived from the breakpoint region of *FGFR2-TDRD1* fusions. Furthermore, CD4^+^ T cells and their T-cell receptors that possess this specific recognition ability can be successfully isolated (59). This finding confirms the immunogenicity of *FGFR2* fusions, providing a theoretical basis for utilizing TILs to target oncogenic fusions. In clinical practice, a case report involved a patient with metastatic cholangiocarcinoma. The patient received a TIL infusion, where about 25% of the T cells were reactive to an *ERBB2IP* mutation. Following this treatment, the patient experienced sustained tumor stabilization and partial regression for over one year. Notably, when the disease later progressed, a second infusion was given. This second infusion contained a highly purified (>95%) population of mutation-specific TILs. The result was again tumor shrinkage, demonstrating the durable anti-tumor activity of TILs in BTC (60). To optimize TIL therapy, a research team established an orthotopic mouse model of CCA to systematically evaluate two expansion strategies: traditional CD3 agonist-based expansion and tumor antigen-driven expansion. Results showed that while both methods effectively expanded TILs, the tumor antigen-driven expansion method demonstrated stronger cytotoxicity *in vitro*, maintaining high killing activity even at low effector:target ratios. Meanwhile, the persistence of these TILs was also significantly superior to those expanded by the traditional method *in vivo* (61).

Recent strategies based on modifying peripheral blood-derived T cells offer new directions for TIL therapy. One study proposed a “three-in-one” strategy, creating super circulating TIL-like cells (ScTILs) for treating advanced BTC. In the corresponding clinical trial, ScTILs demonstrated a manageable safety profile with no severe cytokine release syndrome (CRS) or neurotoxicity observed, which was likely attributable to the omission of both lymphodepleting chemotherapy and high-dose IL-2 support. In patients with normal baseline B-cell levels receiving an appropriate dose, the median overall survival reached 18.3 months, superior to existing standard treatment options (62). This strategy provides a new approach to overcome limitations of traditional TIL therapy regarding cell source, production timeline, and *in vivo* persistence. TILs hold significant value in the integrated diagnosis and treatment of BTC. As key components of the TME, their characteristics provide important insights for efficacy prediction and prognosis assessment (63). Meanwhile, TIL-based adoptive cell therapy is opening a promising innovative treatment pathway for advanced patients.

## 4 Natural killer cell therapy

Natural killer cells are crucial components of the innate immune system, capable of recognizing and killing tumor cells without the need for antigen-specific priming. NK cell activity is controlled by the balance between inhibitory receptors and activating receptors on their surface. Importantly, their function is not restricted by the major histocompatibility complex (MHC). In cancer immunotherapy, NK cells attract considerable attention due to their broad anti-tumor activity and relatively low risk of toxicity. NK cells primarily kill tumor cells and promote anti-tumor immunity by releasing perforin/granzymes, expressing Fas ligand, mediating antibody-dependent cellular cytotoxicity (ADCC), and secreting pro-inflammatory cytokines (64–66). NK cells currently used for adoptive immunotherapy are mainly derived from autologous or allogeneic peripheral blood, umbilical cord blood, or the NK-92 cell line. NK cells derived from induced pluripotent stem cells (iPSC-derived NK cells), leveraging the core advantage of being “off-the-shelf” cell products, offer a novel strategy to address these bottlenecks. Recent research has successfully developed feeder-free monolayer culture systems capable of efficiently inducing high-purity, functional iPSC-derived NK cells (iNK cells). Transcriptomic analysis shows that iNK cells closely resemble peripheral blood-derived NK cells at the gene expression level. In functional assays, iNK cells effectively kill cholangiocarcinoma cell lines, such as KKU-055 and KKU-213A, in both 2D and 3D tumor spheroid models. Their cytotoxic ability is greater than that of the NK-92 cell line, making them a superior cell source for clinical use in cancer immunotherapy (67).

Preclinical studies confirm that ex vivo expanded NK cells can significantly inhibit tumor growth in CCA xenograft models, with multiple infusions not eliciting significant toxicity (68). Comparative studies showed that NK cells exhibited stronger direct killing activity in short-term cytotoxicity assays compared to Vδ2 γδ T cells. Particularly noteworthy, the combination with an anti-EGFR monoclonal antibody significantly enhanced NK cell-mediated ADCC (69). Engineered NK cells demonstrate potential for precise targeted therapy. CAR-NK cells targeting cellular-mesenchymal epithelial transition factor (cMET) showed specific killing activity against cMET-high CCA cell lines (>80% cell death) but no significant effect on cMET-low cells (70). Recently, novel physical regulation methods have further expanded NK cell application prospects. For example, piezoelectric nanoparticles targeting NK cells (αCD56-P@BT/αNK1.1-P@BT) can generate mechanical and electrical signals under ultrasound stimulation, effectively enhancing NK cell migration, tumor infiltration, and killing performance by activating TRP ion channel-mediated calcium influx and cytoskeleton rearrangement. In a mouse model of iCCA, NK cells loaded with these nanoparticles exhibited excellent anti-tumor effects (71). Clinically, Leem et al. conducted a phase I/IIa trial evaluating the safety and efficacy of allogeneic NK cells combined with the PD-1 inhibitor Pembrolizumab in patients with chemotherapy-refractory advanced BTC. Results showed no direct drug-related serious adverse events in the combination therapy. The overall response rate (ORR) in the per-protocol set reached 50.0%, with a disease control rate (DCR) of 62.5% and a median PFS of 4.1 months. These outcomes are significantly better than historical data for Pembrolizumab monotherapy (72). This study provided the first clinical validation of the synergistic anti-tumor potential of NK cells combined with immune checkpoint inhibitors in BTC. In summary, based on optimized NK cell expansion techniques and the development of combination anti-tumor strategies, NK cell therapy shows broad prospects in BTC.

## 5 Cytokine-induced killer cell therapy

Cytokine-induced killer cells are a heterogeneous group of immune effector cells. Their main component is CD3^+^CD56^+^ NKT-like cells. These cells combine the strong ability to proliferate outside the body from T cells with the non-MHC-restricted killing function of NK cells. These cells can be efficiently induced and massively expanded from human peripheral blood mononuclear cells, bone marrow, or umbilical cord blood-derived mononuclear cells by sequential addition of IFN-γ, anti-CD3 monoclonal antibody, and high-dose recombinant human IL-2 (73). CIK cells primarily induce tumor cell apoptosis via the granzyme-perforin pathway, and their cytotoxicity depends on the NKG2D receptor recognizing stress-related ligands like MICA/B expressed on tumor cell surfaces (74, 75). Beyond direct tumor cell killing, CIK cells can also modulate immune responses by secreting various cytokines (e.g., IL-2, IFN-γ, TNF-α, IL-4) (76).

In severe combined immunodeficient (SCID) mouse human CCA xenograft models, CIK cells demonstrated the ability to specifically infiltrate tumor tissue and effectively inhibit tumor growth. Further research indicated that co-culturing CIK cells with dendritic cells (DCs) could enhance their anti-tumor activity, while using purified CD3^+^CD56^+^ subsets could avoid the immunosuppressive effects induced by tumor RNA-pulsed DCs (77). The research team led by Morisaki conducted two independent studies exploring synergistic killing mechanisms. These studies focused on cytokine-activated killer (CAK) lymphocytes in combination with different drugs. In an *in vitro* study on CCA, combining CAK cells with cetuximab significantly enhanced the killing of CCA cell lines via ADCC (78). In a model of chemotherapy-resistant metastatic solid tumors, pretreatment with gemcitabine increased the expression of MICA/B on tumor cell surfaces. This upregulation enhanced CAK cell cytotoxicity through the NKG2D receptor (79). Together, these two studies indicate that combining CAK cells with either targeted drugs or chemotherapy agents can synergistically enhance anti-tumor effects through distinct immune mechanisms. Furthermore, studies show that overexpressing the inducible T-cell costimulator (ICOS) in CIK cells significantly improves their proliferation and cytokine secretion, particularly production of IFN-γ. This enhancement increases their killing activity against BTC cells (80, 81). When ICOS binds to its ligand (ICOSL) on tumor cells, it activates the phosphatidylinositol 3-kinase/protein kinase B and extracellular signal-regulated kinase signaling pathways. This activation promotes CIK cell survival and strengthens their anti-apoptotic ability, thereby improving their persistence and cytotoxicity within the tumor microenvironment.

Clinical research data support the safety and preliminary efficacy of CIK/CAK cells in BTC treatment. A study reported that among 5 patients with chemotherapy-resistant metastatic BTC receiving gemcitabine combined with CAK therapy, 2 achieved a complete response and 3 achieved stable disease, with overall survival ranging from 16 to 64 months. Importantly, no severe adverse events related to cell infusion were observed, suggesting a favorable safety profile (79). Furthermore, a case report detailed a patient with iCCA whose disease progressed after bi-specific antibody conjugated CIK cell therapy. This patient subsequently achieved a complete response after receiving a PD-1 inhibitor. In this case, the sequential strategy was well-tolerated with no reported severe adverse events, suggesting that CIK therapy may effectively prime the immune microenvironment for subsequent checkpoint blockade without compounding toxicity (82). These findings suggest that CIK cell therapy can effectively inhibit BTC progression, and its combination with other therapies represents an important future direction for anti-cancer treatment. However, attention must also be given to potential adverse reactions, such as cytokine release syndrome and autoimmune diseases, which may be more pronounced in combination therapies. The safety profile of these combination approaches needs thorough evaluation alongside efficacy assessments.

## 6 T-cell receptor-engineered T-cell therapy

TCR-T therapy involves genetically reprogramming a patient’s T cells. This process equips them with a new T-cell receptor that can identify specific tumor antigens presented by MHC molecules on cancer cells. Upon recognition, the engineered T cells effectively eliminate the tumor. In contrast to CAR-T therapy, which is confined to targeting surface antigens, TCR-T technology can detect antigens derived from a tumor’s intracellular proteins. This capability significantly expands the range of targetable tumors and positions TCR-T as a highly promising strategy for treating solid tumors (83). Nonetheless, the application of this innovative therapy in BTC is still in early exploration, with early-phase trials including NCT05194735.

Cancer-testis antigens (CTAs) are considered ideal targets for immunotherapy. This is because they are specifically expressed in various cancers, but in normal tissues, their presence is largely restricted to immune-privileged sites such as the testis and placenta (84, 85). Data show that in about 33.7% of iCCA cases, CTAs and HLA class I molecules are co-expressed (86). This co-expression creates a functional basis for TCR-T cells to recognize and attack tumor cells. Studies targeting New York esophageal squamous cell carcinoma 1 (NY-ESO-1) have further strengthened the case for CTA-directed therapy. In both preclinical and clinical settings, NY-ESO-1-specific TCR-T cells strongly suppressed localized and disseminated tumors (87–89). Another promising CTA in iCCA is melanoma-associated antigen (MAGE) A3. Its expression increases gradually during the transition from biliary epithelial dysplasia to invasive carcinoma, indicating it may play a role in cancer development. This dynamic expression also offers a pathological rationale for targeting MAGE-A3 in immunotherapy (86). In TCR-T development, high-affinity TCRs targeting the MAGE*-A3*:112–120 epitope were successfully screened using an HLA-A*02:01 transgenic mouse model. These TCRs could not only recognize MAGE-A3-derived peptides but also cross-react with homologous antigens like MAGE-A12, MAGE-A2, and MAGE-A6 (90). This cross-reactivity significantly broadens their potential targeting range.

Alpha-fetoprotein (AFP) is a well-established clinical biomarker for hepatocellular carcinoma (HCC). Its specificity and high expression levels in HCC patients make it an ideal target for TCR-based therapy. Given that some iCCA cases also express AFP, this strategy could potentially be extended to AFP-positive iCCA patients. Existing evidence indicates that AFP is not only highly expressed in HCC but also detected in a subset of iCCA tumor cells, and these AFP-positive cells exhibit cancer stem cell properties like self-renewal and high tumorigenicity (91). Multiple studies confirm that TCR-T cells can specifically recognize and kill AFP-positive tumor cells, further supporting the feasibility of this strategy in iCCA (92–94).

In BTC, driver mutations exhibit significant heterogeneity across anatomical subtypes. Genomic analyses indicate that *TP53* (26%) and *KRAS* (18%) are among the most prevalent alterations (95). Notably, *KRAS* mutations are enriched in extrahepatic cholangiocarcinoma and gallbladder cancer compared to intrahepatic cases. This distinct mutational landscape underscores the particular promise of TCR-T therapies targeting these shared neoantigens for subsets of patients, especially those with extrahepatic disease. Exemplifying this approach, the Phase I/II trial NCT05194735 engineers autologous T cells via a non-viral Sleeping Beauty transposon/transposase system to target specific *KRAS* and *TP53* mutations. Preliminary reports suggest these TCR-T products can be successfully manufactured and administered without dose-limiting toxicities, providing early evidence for the feasibility and safety of targeting intracellular driver mutations through an MHC-restricted mechanism (96).

In summary, antigens such as MAGE-A3, AFP, and KARS have been detected in a subset of biliary tract cancers. Targeting these antigens with TCR-T therapy has already shown promising preclinical and clinical results in other cancer types, providing a strong rationale and a translational foundation for further investigating their use in BTC.

## 7 Challenges and optimization strategies

Adoptive cell immunotherapy is a key branch of cancer treatment with broad potential for biliary tract cancers. This field includes several approaches, such as CAR-T, TIL, CIK, NK, and TCR-T therapies, each with its own distinct features. Recent advances in immunology and genetic engineering have accelerated both basic and clinical research in this area. Meanwhile, multiple clinical trials are investigating adoptive cell immunotherapy for biliary tract cancers (**Table 2**). Despite this progress, the clinical application for BTC remains challenging due to its complex tumor biology and microenvironment. Addressing these hurdles will demand innovative, multi-level solutions that integrate knowledge across different fields.

**Table 2:** Summary of clinical trials for the adoptive cell immunotherapy of BTC.

Trial	Phase	Status	Interventions	Target	Primary outcomes
NCT03633773	Phase 1/2	Unknown status	CAR-T	MUC-1	DCR
NCT06043466	Phase 1	Recruiting	CAR-T	CEA	Dose range, DLT, MTD
NCT06126406	Phase 1	Recruiting	CAR-T	CEA	AE, DLT
NCT06010862	Phase 1	Recruiting	CAR-T	CEA	AE, MTD
NCT06196658	Phase 1	Not yet recruiting	CAR-T	EX02	AE, ORR
NCT01869166	Phase 1/2	Unknown status	CAR-T	EGFR	AE
NCT01935843	Phase 1/2	Unknown status	CAR-T	HER-2	AE
NCT04660929	Phase 1	Active	CAR-macrophage	HER-2	AE, Feasibility of manufacturing CT-0508
NCT03801083	Phase 2	Recruiting	TIL	NA	ORR
NCT01174121	Phase 2	Recruiting	TIL	NA	ORR
NCT04426669	Phase 1/2	Active	TIL	NA	MTD, AE, ORR
NCT02482454	Phase 2/3	Active	CIK	NA	RFS
NCT01868490	Phase 1/2	Enrolling by invitation	CIK	NA	Tumor size, CIK cell-homing, FACS analysis
NCT03358849	Phase 1	Completed	NK	NA	DLT, MTD
NCT03937895	Phase 1/2	Completed	NK	NA	DLT, ORR
NCT05976906	Phase 1	Unknown status	NK	NA	AE, DLT, MTD
NCT05194735	Phase 1/2	Terminated	TCR-T	NA	AE, DLT, MTD

NA, not available; DCR, Disease control rate; DLT, Dose-limiting toxicity; MTD, Maximum tolerable dose; AE, Adverse Events; ORR, Objective response rate; RFS, Recurrence-free survival; FACS, Fluorescence-activated cell sorting.

The TME of BTC is highly heterogeneous and immunosuppressive. Its structure is built upon a dense extracellular matrix and various stromal cells. It is also populated by immunosuppressive cells, including M2 macrophages and Tregs, and filled with factors like TGF-β and IL-10 (97–99). These components create a powerful network that blocks drug delivery and weakens immune cell function. Consequently, this promotes tumor progression and metastasis. Research shows that only a tiny fraction of therapeutic T cells successfully reach the tumor core, and this limited infiltration greatly reduces treatment effectiveness (100).

Beyond the hindrances of the TME, effector cells themselves face significant functional deficiencies and survival challenges. CAR-T cells often fail to achieve long-term persistence *in vivo* due to insufficient support from key cytokines and rejection by the host immune system (101). In addition, CAR-T cells are prone to exhaustion, especially after prolonged exposure to tumors. This exhausted state is marked by high levels of inhibitory receptors. Studies using 3D tumor models show that CAR-T cells can lose their tumor-killing ability relatively quickly after becoming exhausted (52). TIL and CIK therapies contain mixed populations of cells, and not all of these cells contribute to fighting the tumor. Some may even suppress the activity of other immune cells (102, 103). Meanwhile, NK cells are highly sensitive to the process of cryopreservation and thawing, often suffering a substantial loss in both viability and killing activity. This sensitivity currently restricts their clinical effectiveness (104).

Target-related challenges also pose major obstacles for adoptive cell therapy. Tumor cells can evade immune attack by downregulating HLA molecules or target antigens, causing immunotherapies to lose their target. This is known as antigen escape (105, 106). The high heterogeneity of BTC further complicates treatment. The expression of key targets often varies significantly between patients and even between different tumor sites in the same individual (95, 107, 108). Another critical issue is “on-target, off-tumor” toxicity. If the target antigen is also present on normal tissues, the therapeutic cells may attack healthy cells, causing serious side effects (109). For instance, the cMET antigen is expressed in about 50%-60% of CCA patients, making it a potential target (110). However, because cMET is also found in normal bile ducts and other tissues, targeting it carries a risk of severe damage to these healthy organs, directly threatening patient safety (70).

Each technological pathway also has its specific bottlenecks. Traditional CAR-T cells lack effective safety switches and are prone to significant exhaustion in environments with high PD-L1 expression, which can lead to severe CRS (111, 112). The TIL preparation process is complex, time-consuming, and difficult to standardize. It requires high-dose IL-2 for expansion, and the T cells are prone to exhaustion during this process (113, 114). Furthermore, obtaining sufficient tumor tissue can be a major barrier for advanced patients. For CIK cell therapy, a key drawback is that IL-2, while crucial for inducing CIK cells, also promotes the expansion of Tregs. This can potentially counteract the anti-tumor effect (115, 116). NK cell therapy, on the other hand, faces source limitations. NK cells derived from peripheral or cord blood are limited in number and heterogeneity, and iPSC differentiation technology is not yet fully mature. TCR-T technology is strictly limited by HLA restriction. Its efficacy is dependent on matching specific HLA types, limiting the broad application of TCR-T cells (117, 118).

To address these challenges, researchers have proposed various innovative strategies, primarily focusing on three dimensions: genetic engineering, optimization of combination therapies, and innovations in manufacturing processes. In genetic engineering, modifying the CAR structure through approaches like converting inhibitory signals or blocking multiple inhibitory receptors can significantly enhance CAR-T cell tumor-killing capacity. For example, one approach uses a PD-1–CD28 switch receptor. When this modified CAR-T cell encounters PD-L1, it turns the inhibitory signal into a stimulating one. In experiments, these cells achieved a 70.69% killing rate against PD-L1–high BTC cells and showed reduced exhaustion markers (35). Complementing receptor modification, gene editing via CRISPR/Cas9 enables the precise disruption of inhibitory pathways. One strategy involves targeting multiple immunosuppressive molecules, such as PD-1 and receptors for cytokines like TGF-β, IL-10, and IL-6. This approach has been shown to generate stronger CAR-T cells that demonstrate improved tumor infiltration and increased IFN-γ secretion. In humanized cholangiocarcinoma models, this ultimately leads to significant tumor regression and prolonged survival (119). Separately, armored CAR-T or TCR-T cells are engineered to remodel the immunosuppressive tumor microenvironment. They secrete pro-inflammatory cytokines, which enhance T-cell activity and attract endogenous immune cells. Novel approaches now use endogenous tumor-specific promoters to confine payload delivery to the tumor site. This localized release reduces systemic toxicity and improves the balance between treatment strength and safety (120–122). Concurrently, safety mechanisms such as “off-switches” or suicide genes have been introduced to induce CAR elimination if needed (123, 124). Meanwhile, developing dual-target CAR structures and sequential treatment strategies can effectively counter antigen escape by tumor cells. These approaches expand target recognition to include multiple antigens, enhancing coverage of heterogeneous tumors and reducing the risk of relapse due to antigen loss (39, 125). Beyond expanding the target repertoire, further engineering sophistication is aimed at enhancing the precision of tumor recognition. Although not yet reported in BTC, logic-gated CAR systems represent a highly promising strategy to overcome heterogeneity while minimizing on-target, off-tumor toxicity. These systems require the presence (or absence) of multiple tumor-associated antigens for full T-cell activation, thereby theoretically confining activity to tumor cells expressing the exact antigen combination while sparing normal tissues that may express only one (126). The successful application of such strategies in other solid tumors provides a solid foundation for their exploration given BTC’s complex antigenic profile. Collectively, these next-generation engineering strategies aim to not only enhance the intrinsic fitness of effector cells against exhaustion but also dynamically reshape the immunosuppressive environment and adapt to the heterogeneous landscape of biliary tract cancer.

In combination therapy strategies, the combination of immune checkpoint inhibitors with adoptive cell therapy shows synergistic effects and has received empirical support in treating various malignancies (127–129). In CCA research, the combination of B7-H3 CAR-T cells with PD-1 blockade significantly enhanced tumor clearance in patient-derived tumor spheroid models (52). On the other hand, combining adoptive cell therapy with radiotherapy or chemotherapy can induce a more comprehensive anti-tumor immune response. Studies indicate that low-dose radiotherapy can optimize the TME by inducing iNOS+/M1-type macrophage phenotypic reprogramming while upregulating chemokine secretion, thereby synergistically potentiating the tumor infiltration and anti-tumor activity of adoptive T cells (130, 131). Chemotherapeutic drugs like gemcitabine can induce immunogenic cell death, promoting the exposure and presentation of tumor antigens. Thus, integrating adoptive cell therapy with modalities like checkpoint blockade, radiotherapy, or chemotherapy constitutes a powerful therapeutic approach by leveraging their different mechanisms of action (79).

**Figure 2:**
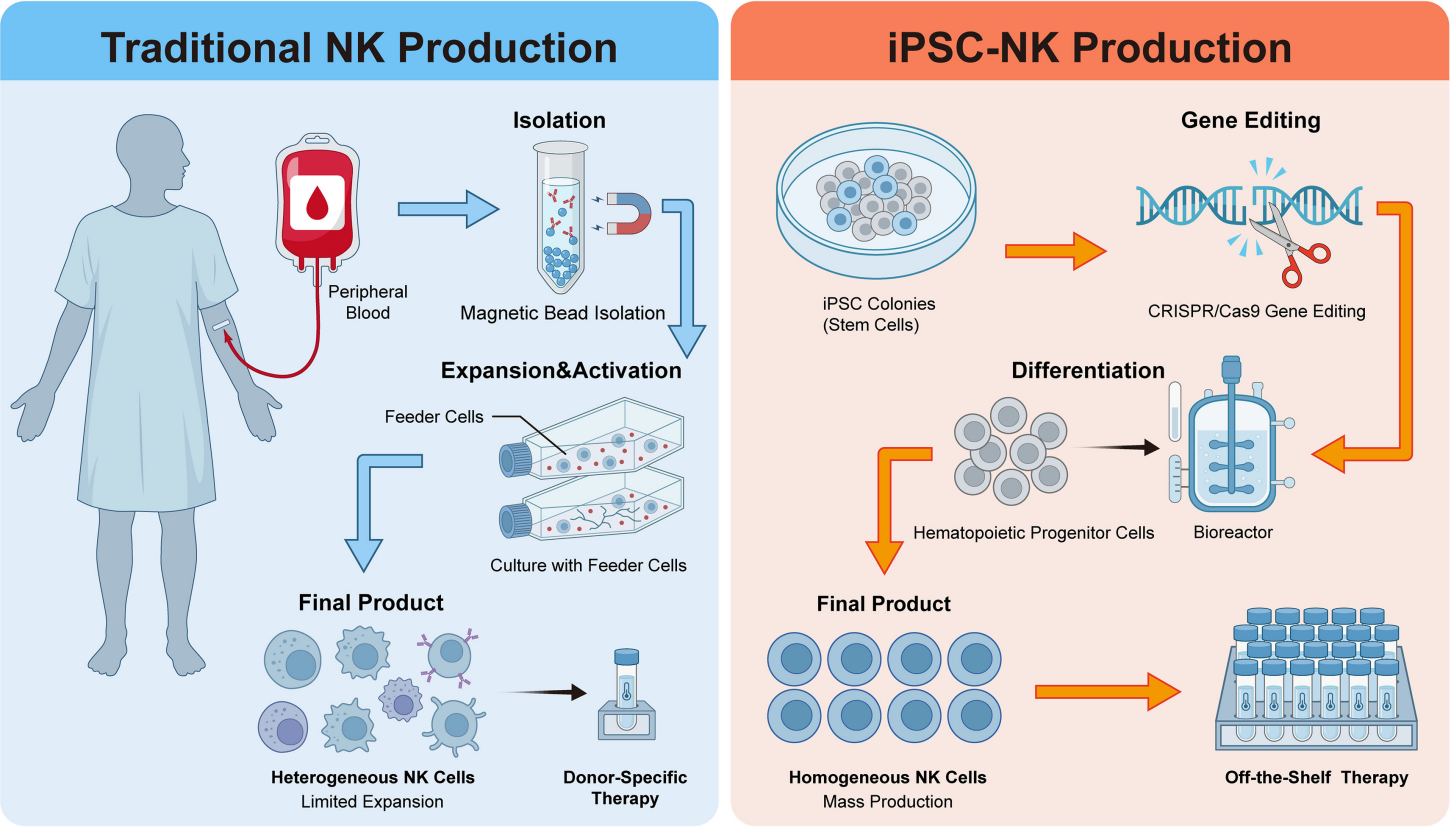
Comparison of traditional and iPSC-based NK cell production strategies. The left panel illustrates the traditional approach, involving isolation from donor peripheral blood and expansion with feeder cells, resulting in a heterogeneous final product. The right panel depicts the iPSC-NK production workflow, which utilizes CRISPR/Cas9 gene editing and bioreactor-based differentiation to generate homogeneous, off-the-shelf NK cell therapies.

Innovations in manufacturing processes and product forms have also made significant progress. In the NK cell field, advances in iPSC differentiation technology make the large-scale production of “off-the-shelf” NK cell products possible (**Figure 2**). Feeder-free monolayer culture systems can yield high-purity, highly cytotoxic iPSC-derived NK cells, whose cytotoxicity is superior to the traditional NK-92 cell line (67, 132). In the T-cell therapy field, the development of ScTILs represents an innovative strategy. Instead of extracting cells from tumor tissue, ScTILs are isolated from a patient’s blood. These PD-1-positive T cells are genetically engineered using lentiviral vectors to express an enhanced receptor that reverses immunosuppressive signals from the tumor microenvironment. A CD19-targeting CAR is also introduced, enabling rapid expansion in the body via B-cell activation (62). Unlike traditional TIL therapy, ScTILs do not require tumor resection, lymphodepleting chemotherapy, or high-dose IL-2. This results in shorter production time, improved safety, and broader accessibility. Clinical studies in advanced BTC have shown significant survival benefits with this approach. Another emerging modality is TCR-like antibodies. These antibodies can specifically recognize peptide-MHC complexes on tumor cells and exert anti-tumor effects through several mechanisms: they can be engineered into CAR-T cells, mediate effector functions like ADCC, or directly trigger tumor cell apoptosis. Unlike TCR-T therapy, TCR-like antibodies do not require complex cell manipulation or patient-specific expansion. They can be used as “off-the-shelf” products, broadening the potential of cellular immunotherapy (133, 134). The introduction of nanotechnology further enhances the efficacy and safety of adoptive cell therapy. Nanocarriers enable targeted delivery of gene editing tools, RNA, or cytokines. This approach improves immune cell activation, function, and persistence *in vivo*, while helping to modulate the immunosuppressive tumor microenvironment (135–138). Simultaneously, nanotechnology can also serve as a novel regulation method, providing new technical means for precisely controlling effector cell function (71, 139).

Emerging strategies are looking beyond directly targeting cancer cells. Instead, they focus on remodeling the non-tumor components of the TME, such as cancer-associated fibroblasts, macrophages, and dendritic cells, and disrupting their immunosuppressive interaction networks. Cancer-associated fibroblasts that express fibroblast activation protein are particularly important in iCCA. They not only build a dense scar-like barrier but also orchestrate a suppressive network by secreting chemokines. Specifically, the signaling axis involving signal transducer and activator of transcription 3 and C-C motif chemokine ligand 2 actively recruits myeloid-derived suppressor cells, while C-X-C motif chemokine ligand 12 and its receptor contribute to the exclusion of effector T cells (140, 141). Preclinical studies show that CAR-T cells designed to target fibroblast activation protein can successfully remove these supportive cells. This depletion severs the communication link between cancer-associated fibroblasts and myeloid-derived suppressor cells, breaks down the physical barrier and makes tumors more responsive to subsequent treatment with tumor-targeting CAR-T cells (142, 143). Parallel to stromal depletion, exploiting the plasticity of myeloid cells represents another prospective avenue. Tumor-associated macrophages typically maintain an immunosuppressive M2 phenotype through crosstalk with tumor cells via the PD-1/PD-L1 axis (144). Chimeric antigen receptor macrophages are a new tool that can penetrate dense tumors and reverse this polarity. Beyond mediating phagocytosis, CAR-macrophage cells secrete pro-inflammatory cytokines that repolarize the local niche from M2 to M1 (145–147). A phase I trial (NCT04660929) evaluating anti-HER2 CAR-macrophages is currently underway, offering a translational precedent for HER2-positive BTC. Furthermore, effective anti-tumor immunity relies on the interplay between T cells and antigen-presenting cells, which is often disrupted in the TME. Engineered T cells secreting Fms-like tyrosine kinase 3 ligand and lymphotactin have been shown to actively recruit and activate conventional type 1 dendritic cells. This restored interaction between dendritic cells and T cells promotes robust antigen spreading and endogenous T cell clonal expansion, effectively counteracting tumor heterogeneity (148). These multi-pronged strategies are therefore particularly promising for the treatment of BTC. The tumor microenvironment in BTC is typically highly complex, fibrotic, and immunosuppressive. By targeting these critical cellular nodes and their communication links, this integrated approach aims to transform this hostile setting into one that enables sustained anti-tumor immunity.

Future breakthroughs in adoptive cell therapy for BTC will require a smart and combined use of the strategies we have discussed. The next generation of treatments will likely not rely on a single type of engineered cell. Instead, they will form a living drug system that can dynamically adapt to BTC’s complex tumor microenvironment. This approach promises to overcome key challenges like immunosuppression, T-cell exhaustion, and target heterogeneity. Ultimately, it could provide BTC patients with safer and more durable treatment options.

## 8 Summary

The treatment of biliary tract cancer faces severe challenges due to its high heterogeneity and immunosuppressive microenvironment. Adoptive cell therapies, including CAR-T, TIL, NK, CIK, and TCR-T, offer new hope for advanced patients by activating or engineering immune cells to precisely target tumors. Although showing potential in target selection and clinical research, this field remains constrained by key bottlenecks such as TME suppression, limited effector cell infiltration and functional exhaustion, target heterogeneity, and off-tumor toxicity. Future breakthroughs depend on strategies like genetic engineering and combination with immune checkpoint inhibitors to synergistically overcome these barriers, thereby promoting clinical translation and improving patient outcomes.

## References

[1] Perez-Moreno P Riquelme I Garcia P Brebi P Roa JC . Environmental and lifestyle risk factors in the carcinogenesis of gallbladder cancer. J Pers Med. (2022) 12:234. doi: 10.3390/jpm12020234, PMID: 35207722 PMC8877116

[2] Qurashi M Vithayathil M Khan SA . Epidemiology of cholangiocarcinoma. Eur J Surg Oncol. (2025) 51:107064. doi: 10.1016/j.ejso.2023.107064, PMID: 37709624

[3] Su J Liang YH He XF . Global, regional, and national burden and trends analysis of gallbladder and biliary tract cancer from 1990 to 2019 and predictions to 2030: A systematic analysis for the global burden of disease study 2019. Front Med. (2024) 11:1384314. doi: 10.3389/fmed.2024.1384314, PMID: 38638933 PMC11024434

[4] Wen WQ Mumma M Zheng W . Temporal trends of stages and survival of biliary tract cancers in the United States and associations with demographic factors. Cancer Epidemiol Biomarkers Prev. (2023) 32:1660–7. doi: 10.1158/1055-9965.Epi-23-0562, PMID: 37606709 PMC10840886

[5] Wilbur HC Soares HP Azad NS . Neoadjuvant and adjuvant therapy for biliary tract cancer: advances and limitations. Hepatology. (2025) 82:1287–302. doi: 10.1097/hep.0000000000000760, PMID: 38266282

[6] Valle JW Kelley RK Nervi B Oh DY Zhu AX . Biliary tract cancer. Lancet. (2021) 397:428–44. doi: 10.1016/S0140-6736(21)00153-7, PMID: 33516341

[7] Valle J Wasan H Palmer DH Cunningham D Anthoney A Maraveyas A . Cisplatin plus gemcitabine versus gemcitabine for biliary tract cancer. N Engl J Med. (2010) 362:1273–81. doi: 10.1056/NEJMoa0908721, PMID: 20375404

[8] Raggi C Taddei ML Rae C Braconi C Marra F . Metabolic reprogramming in cholangiocarcinoma. J Hepatol. (2022) 77:849–64. doi: 10.1016/j.jhep.2022.04.038, PMID: 35594992

[9] Aliazis K Christofides A Shah R Yeo YY Jiang S Charest A . The tumor microenvironment’s role in the response to immune checkpoint blockade. Nat Cancer. (2025) 6:924–37. doi: 10.1038/s43018-025-00986-3, PMID: 40514448 PMC12317369

[10] Liu Y Zhang X Gu W Su H Wang X Wang X . Unlocking the crucial role of cancer-associated fibroblasts in tumor metastasis: mechanisms and therapeutic prospects. J Adv Res. (2025) 71:399–413. doi: 10.1016/j.jare.2024.05.031, PMID: 38825314 PMC12126706

[11] Alsaafeen BH Ali BR Elkord E . Resistance mechanisms to immune checkpoint inhibitors: updated insights. Mol Cancer. (2025) 24:20. doi: 10.1186/s12943-024-02212-7, PMID: 39815294 PMC11734352

[12] Vogel A Ducreux M . Esmo clinical practice guideline interim update on the management of biliary tract cancer. ESMO Open. (2025) 10:104003. doi: 10.1016/j.esmoop.2024.104003, PMID: 39864891 PMC11846563

[13] Casak SJ Kumar V Song C Yuan M Amatya AK Cheng J . Fda approval summary: durvalumab and pembrolizumab, immune checkpoint inhibitors for the treatment of biliary tract cancer. Clin Cancer Res. (2024) 30:3371–7. doi: 10.1158/1078-0432.Ccr-24-0517, PMID: 38856639 PMC11326973

[14] Demir T Moloney C Mahalingam D . Emerging targeted therapies and strategies to overcome resistance in biliary tract cancers. Crit Rev Oncol Hematol. (2024) 199:104388. doi: 10.1016/j.critrevonc.2024.104388, PMID: 38754771

[15] Zhou T Wu Y Li S Qiu X Liu E Xie Z . Multi-omic analysis of gallbladder cancer identifies distinct tumor microenvironments associated with disease progression. Nat Genet. (2025) 57:1935–49. doi: 10.1038/s41588-025-02236-9, PMID: 40571730

[16] Li YQ Zheng YT Liu TQ Liao CY Shen GB He ZY . The potential and promise for clinical application of adoptive T cell therapy in cancer. J Transl Med. (2024) 22:413. doi: 10.1186/s12967-024-05206-7, PMID: 38693513 PMC11064426

[17] Kalos M Levine BL Porter DL Katz S Grupp SA Bagg A . T cells with chimeric antigen receptors have potent antitumor effects and can establish memory in patients with advanced leukemia. Sci Transl Med. (2011) 3:95ra73. doi: 10.1126/scitranslmed.3002842, PMID: 21832238 PMC3393096

[18] Qi C Liu C Gong J Liu D Wang X Zhang P . Claudin18.2-specific car T cells in gastrointestinal cancers: phase 1 trial final results. Nat Med. (2024) 30:2224–34. doi: 10.1038/s41591-024-03037-z, PMID: 38830992

[19] Pinto N Albert CM Taylor MR Ullom HB Wilson AL Huang W . Strive-02: A first-in-human phase I study of systemically administered B7-H3 chimeric antigen receptor T cells for patients with relapsed/refractory solid tumors. J Clin Oncol. (2024) 42:4163–72. doi: 10.1200/jco.23.02229, PMID: 39255444

[20] Min J Long C Zhang L Duan J Fan H Chu F . C-met specific car-T cells as a targeted therapy for non-small cell lung cancer cell A549. Bioengineered. (2022) 13:9216–32. doi: 10.1080/21655979.2022.2058149, PMID: 35378051 PMC9161852

[21] Myers RM Devine K Li Y Lawrence S Leahy AB Liu H . Reinfusion of cd19 car T cells for relapse prevention and treatment in children with acute lymphoblastic leukemia. Blood Adv. (2024) 8:2182–92. doi: 10.1182/bloodadvances.2024012885, PMID: 38386999 PMC11061218

[22] Zhang XM Zhang H Lan HX Wu JM Xiao Y . Car-T cell therapy in multiple myeloma: current limitations and potential strategies. Front Immunol. (2023) 14:1101495. doi: 10.3389/fimmu.2023.1101495, PMID: 36891310 PMC9986336

[23] Kwon N Chen YY . Overcoming solid-tumor barriers: armored car-T cell therapy. Trends Cancer. (2025) 11:1019–29. doi: 10.1016/j.trecan.2025.08.009, PMID: 40940282

[24] Baker DJ Arany Z Baur JA Epstein JA June CH . Car T therapy beyond cancer: the evolution of a living drug. Nature. (2023) 619:707–15. doi: 10.1038/s41586-023-06243-w, PMID: 37495877 PMC12522170

[25] Kagoya Y Tanaka S Guo TX Anczurowski M Wang CH Saso K . A novel chimeric antigen receptor containing a jak-stat signaling domain mediates superior antitumor effects. Nat Med. (2018) 24:352–9. doi: 10.1038/nm.4478, PMID: 29400710 PMC5839992

[26] Dejenie TA Medhin MTG Terefe GD Admasu FT Tesega WW Abebe EC . Current updates on generations, approvals, and clinical trials of car T-cell therapy. Hum Vaccin Immunother. (2022) 18:2114254. doi: 10.1080/21645515.2022.2114254, PMID: 36094837 PMC9746433

[27] Zheng ZB Li SY Liu MH Chen CY Zhang L Zhou DB . Fine-tuning through generations: advances in structure and production of car-T therapy. Cancers. (2023) 15:3476. doi: 10.3390/cancers15133476, PMID: 37444586 PMC10340266

[28] Radziejewska I . Tumor-associated carbohydrate antigens of muc1-implication in cancer development. BioMed Pharmacother. (2024) 174:116619. doi: 10.1016/j.biopha.2024.116619, PMID: 38643541

[29] Zhang G Zheng G Zhang H Qiu L . Muc1 induces the accumulation of foxp3(+) treg cells in the tumor microenvironment to promote the growth and metastasis of cholangiocarcinoma through the egfr/pi3k/akt signaling pathway. Int Immunopharmacol. (2023) 118:110091. doi: 10.1016/j.intimp.2023.110091, PMID: 37018979

[30] Mall AS Tyler MG Ho SB Krige JEJ Kahn D Spearman W . The expression of muc mucin in cholangiocarcinoma. Pathol Res Pract. (2010) 206:805–9. doi: 10.1016/j.prp.2010.08.004, PMID: 20947262

[31] Mao L Su S Li J Yu SY Gong Y Chen CZ . Development of engineered car T cells targeting tumor-associated glycoforms of muc1 for the treatment of intrahepatic cholangiocarcinoma. J Immunother Cancer. (2023) 46:89–95. doi: 10.1097/cji.0000000000000460, PMID: 36883998 PMC9988215

[32] Supimon K Sangsuwannukul T Sujjitjoon J Phanthaphol N Chieochansin T Poungvarin N . Anti-mucin 1 chimeric antigen receptor T cells for adoptive T cell therapy of cholangiocarcinoma. Sci Rep. (2021) 11:6276. doi: 10.1038/s41598-021-85747-9, PMID: 33737613 PMC7973425

[33] Pyzer AR Stroopinsky D Rosenblatt J Anastasiadou E Rajabi H Washington A . Muc1 inhibition leads to decrease in pd-L1 levels via upregulation of mirnas. Leukemia. (2017) 31:2780–90. doi: 10.1038/leu.2017.163, PMID: 28555079 PMC5791150

[34] Wang R He SW Long J Wang Y Jiang XJ Chen MF . Emerging therapeutic frontiers in cancer: insights into posttranslational modifications of pd-1/pd-L1 and regulatory pathways. Exp Hematol Oncol. (2024) 13:46. doi: 10.1186/s40164-024-00515-5, PMID: 38654302 PMC11040904

[35] Supimon K Sangsuwannukul T Sujjitjoon J Chieochansin T Junking M Yenchitsomanus PT . Cytotoxic activity of anti-mucin 1 chimeric antigen receptor T cells expressing pd-1-cd28 switch receptor against cholangiocarcinoma cells. Cytotherapy. (2023) 25:148–61. doi: 10.1016/j.jcyt.2022.10.006, PMID: 36396553

[36] Kim Y Jee S Kim H Paik SS Choi D Yoo SH . Egfr, her2, and met gene amplification and protein expression profiles in biliary tract cancer and their prognostic significance. Oncologist. (2024) 29:e1051–e60. doi: 10.1093/oncolo/oyae076, PMID: 38709907 PMC11299936

[37] Halder S Basu S Lall SP Ganti AK Batra SK Seshacharyulu P . Targeting the egfr signaling pathway in cancer therapy: what’s new in 2023? Expert Opin Ther Targets. (2023) 27:305–24. doi: 10.1080/14728222.2023.2218613, PMID: 37243489 PMC10330690

[38] Guo YL Feng KC Liu Y Wu ZQ Dai HR Yang QM . Phase I study of chimeric antigen receptor-modified T cells in patients with egfr-positive advanced biliary tract cancers. Clin Cancer Res. (2018) 24:1277–86. doi: 10.1158/1078-0432.Ccr-17-0432, PMID: 29138340

[39] Feng KC Guo YL Liu Y Dai HR Wang Y Lv HY . Cocktail treatment with egfr-specific and cd133-specific chimeric antigen receptor-modified T cells in a patient with advanced cholangiocarcinoma. J Hematol Oncol. (2017) 10:4. doi: 10.1186/s13045-016-0378-7, PMID: 28057014 PMC5217546

[40] Zhang Z Wang Z Liu T Tang J Liu Y Gou T . Exploring the role of itgb6: fibrosis, cancer, and other diseases. Apoptosis. (2024) 29:570–85. doi: 10.1007/s10495-023-01921-6, PMID: 38127283

[41] Brzozowska E Deshmukh S . Integrin alpha V beta 6 (Avβ6) and its implications in cancer treatment. Int J Mol Sci. (2022) 23:12346. doi: 10.3390/ijms232012346, PMID: 36293202 PMC9603893

[42] Sun Q Dong XW Shang YK Sun FK Niu J Li FN . Integrin Avβ6 predicts poor prognosis and promotes resistance to cisplatin in hilar cholangiocarcinoma. Pathol Res Pract. (2020) 216:153022. doi: 10.1016/j.prp.2020.153022, PMID: 32534716

[43] Phanthaphol N Somboonpatarakun C Suwanchiwasiri K Chieochansin T Sujjitjoon J Wongkham S . Chimeric antigen receptor T cells targeting integrin Avβ6 expressed on cholangiocarcinoma cells. Front Oncol. (2021) 11:657868. doi: 10.3389/fonc.2021.657868, PMID: 33763382 PMC7982884

[44] Phanthaphol N Somboonpatarakun C Suwanchiwasiri K Yuti P Sujjitjoon J Augsornworawat P . Enhanced cytotoxicity against cholangiocarcinoma by fifth-generation chimeric antigen receptor T cells targeting integrin Avβ6 and secreting anti-pd-L1 scfv. J Transl Med. (2025) 23:451. doi: 10.1186/s12967-025-06453-y, PMID: 40241132 PMC12004729

[45] Gisina A Kim Y Yarygin K Lupatov A . Can cd133 be regarded as a prognostic biomarker in oncology: pros and cons. Int J Mol Sci. (2023) 24:17398. doi: 10.3390/ijms242417398, PMID: 38139228 PMC10744290

[46] Leelawat K Thongtawee T Narong S Subwongcharoen S Treepongkaruna SA . Strong expression of cd133 is associated with increased cholangiocarcinoma progression. World J Gastroenterol. (2011) 17:1192–8. doi: 10.3748/wjg.v17.i9.1192, PMID: 21448425 PMC3063913

[47] Wei Y Chen Q Chen J Zhou C Geng S Shi D . Loss of A-1,2-mannosidase man1c1 promotes tumorigenesis of intrahepatic cholangiocarcinoma through enhancing cd133-fip200 interaction. Cell Rep. (2023) 42:113588. doi: 10.1016/j.celrep.2023.113588, PMID: 38117655

[48] Sangsuwannukul T Supimon K Sujjitjoon J Phanthaphol N Chieochansin T Poungvarin N . Anti-tumour effect of the fourth-generation chimeric antigen receptor T cells targeting cd133 against cholangiocarcinoma cells. Int Immunopharmacol. (2020) 89:107069. doi: 10.1016/j.intimp.2020.107069, PMID: 33242709

[49] Cheng R Chen YQ Zhou HH Wang B Du Q Chen YL . B7-H3 expression and its correlation with clinicopathologic features, angiogenesis, and prognosis in intrahepatic cholangiocarcinoma. Apmis. (2018) 126:396–402. doi: 10.1111/apm.12837, PMID: 29696716

[50] Zhou WT Jin WL . B7-H3/cd276: an emerging cancer immunotherapy. Front Immunol. (2021) 12:701006. doi: 10.3389/fimmu.2021.701006, PMID: 34349762 PMC8326801

[51] Tan X Zhao XY . B7-H3 in acute myeloid leukemia: from prognostic biomarker to immunotherapeutic target. Chin Med J (Engl). (2024) 137:2540–51. doi: 10.1097/cm9.0000000000003099, PMID: 38595093 PMC11556994

[52] Sun Y Maggs L Panda A Wright SJ Cicerchia AM Jenney A . Tbk1 targeting is identified as a therapeutic strategy to enhance car T-cell efficacy using patient-derived organotypic tumor spheroids. Cancer Immunol Res. (2025) 13:210–28. doi: 10.1158/2326-6066.Cir-23-1011, PMID: 39785827 PMC11790382

[53] Kurokawa T Yamada T Cai L Ferrone S Ferrone CR . B7-H3-specific chimeric-antigen receptor T-cell-based immunotherapy for intrahepatic cholangiocarcinoma. J Am Coll Surg. (2018) 227:S236–S. doi: 10.1016/j.jamcollsurg.2018.07.622

[54] Aizaz M Khan AS Khan M Musazade E Yang G . Advancements in tumor-infiltrating lymphocytes: historical insights, contemporary milestones, and future directions in oncology therapy. Crit Rev Oncol Hematol. (2024) 202:104471. doi: 10.1016/j.critrevonc.2024.104471, PMID: 39117163

[55] Lee H Kim K Chung J Hossain M Lee HJ . Tumor-infiltrating lymphocyte therapy: clinical aspects and future developments in this breakthrough cancer treatment. Bioessays. (2023) 45:e2200204. doi: 10.1002/bies.202200204, PMID: 37166068

[56] Chesney J Lewis KD Kluger H Hamid O Whitman E Thomas S . Efficacy and safety of lifileucel, a one-time autologous tumor-infiltrating lymphocyte (Til) cell therapy, in patients with advanced melanoma after progression on immune checkpoint inhibitors and targeted therapies: pooled analysis of consecutive cohorts of the C-144–01 study. J Immunother Cancer. (2022) 10:e005755. doi: 10.1136/jitc-2022-005755, PMID: 36600653 PMC9748991

[57] Lowery FJ Goff SL Gasmi B Parkhurst MR Ratnam NM Halas HK . Neoantigen-specific tumor-infiltrating lymphocytes in gastrointestinal cancers: A phase 2 trial. Nat Med. (2025) 31:1994–2003. doi: 10.1038/s41591-025-03627-5, PMID: 40169866 PMC12315913

[58] Turcotte S Donia M Gastman B Besser M Brown R Coukos G . Art of til immunotherapy: sitc’s perspective on demystifying a complex treatment. J Immunother Cancer. (2025) 13:e010207. doi: 10.1136/jitc-2024-010207, PMID: 39837618 PMC11752064

[59] White BS Sindiri S Hill V Gasmi B Nah S Gartner JJ . Specific recognition of an fgfr2 fusion by tumor infiltrating lymphocytes from a patient with metastatic cholangiocarcinoma. J Immunother Cancer. (2023) 11:e006303. doi: 10.1136/jitc-2022-006303, PMID: 37045473 PMC10106037

[60] Tran E Turcotte S Gros A Robbins PF Lu YC Dudley ME . Cancer immunotherapy based on mutation-specific cd4+T cells in a patient with epithelial cancer. Science. (2014) 344:641–5. doi: 10.1126/science.1251102, PMID: 24812403 PMC6686185

[61] Wittling MC Bennett FJ Warren EAK Oppat KM Wyatt MM Hammons JN . Development of a murine tumor-infiltrating lymphocyte therapy model for cholangiocarcinoma. J Immunol. (2026) 215(1):vkaf242. doi: 10.1093/jimmun/vkaf242, PMID: 40972018 PMC12704411

[62] Wan X Zhao J Yang X Mou X Liu B Gao B . Therapeutic T cells with 3-in-1 strategy for the treatment of biliary tract cancer. Cell Rep Med. (2025) 6:102349. doi: 10.1016/j.xcrm.2025.102349, PMID: 40972581 PMC12629817

[63] Bang YH Lee CK Bang K Kim HD Kim KP Jeong JH . Artificial intelligence-powered spatial analysis of tumor-infiltrating lymphocytes as a potential biomarker for immune checkpoint inhibitors in patients with biliary tract cancer. Clin Cancer Res. (2024) 30:4635–43. doi: 10.1158/1078-0432.Ccr-24-1265, PMID: 39150517

[64] Myers JA Miller JS . Exploring the nk cell platform for cancer immunotherapy. Nat Rev Clin Oncol. (2021) 18:85–100. doi: 10.1038/s41571-020-0426-7, PMID: 32934330 PMC8316981

[65] Page A Chuvin N Valladeau-Guilemond J Depil S . Development of nk cell-based cancer immunotherapies through receptor engineering. Cell Mol Immunol. (2024) 21:315–31. doi: 10.1038/s41423-024-01145-x, PMID: 38443448 PMC10978891

[66] Vivier E Tomasello E Baratin M Walzer T Ugolini S . Functions of natural killer cells. Nat Immunol. (2008) 9:503–10. doi: 10.1038/ni1582, PMID: 18425107

[67] Thongsin N Suwanpitak S Augsornworawat P Srisantitham J Saiprayong K Jenjaroenpun P . Phenotypic and transcriptomic profiling of induced pluripotent stem cell (Ipsc)-derived nk cells and their cytotoxicity against cancers. Stem Cell Res Ther. (2024) 15:418. doi: 10.1186/s13287-024-04029-z, PMID: 39533434 PMC11559060

[68] Jung IH Kim DH Yoo DK Baek SY Jeong SH Jung DE . *In vivo* study of natural killer (Nk) cell cytotoxicity against cholangiocarcinoma in a nude mouse model. In Vivo. (2018) 32:771–81. doi: 10.21873/invivo.11307, PMID: 29936458 PMC6117784

[69] Kulma I Na-Bangchang K Herrera AC Ndubuisi IT Iwasaki M Tomono H . Analysis of the effector functions of Vδ2 Γδ T cells and nk cells against cholangiocarcinoma cells. Cells. (2024) 13:1322. doi: 10.3390/cells13161322, PMID: 39195212 PMC11352430

[70] Chiawpanit C Wathikthinnakorn M Sawasdee N Phanthaphol N Sujjitjoon J Junking M . Precision immunotherapy for cholangiocarcinoma: pioneering the use of human-derived anti-cmet single chain variable fragment in anti-cmet chimeric antigen receptor (Car) nk cells. Int Immunopharmacol. (2024) 136:112273. doi: 10.1016/j.intimp.2024.112273, PMID: 38810311

[71] Zhang RT Yang WW Zhou ZY Ding MM Wang H Yuan WJ . Ultrasound-activated piezoelectric nanoparticles targeting and activating nk cells for tumor immunotherapy. Adv Mater. (2025) 37(47):e08101. doi: 10.1002/adma.202508101, PMID: 40916189

[72] Leem G Jang SI Cho JH Jo JH Lee HS Chung MJ . Safety and efficacy of allogeneic natural killer cells in combination with pembrolizumab in patients with chemotherapy-refractory biliary tract cancer: A multicenter open-label phase 1/2a trial. Cancers. (2022) 14:4229. doi: 10.3390/cancers14174229, PMID: 36077766 PMC9454779

[73] Pievani A Borleri G Pende D Moretta L Rambaldi A Golay J . Dual-functional capability of cd3+Cd56+ Cik cells, a T-cell subset that acquires nk function and retains tcr-mediated specific cytotoxicity. Blood. (2011) 118:3301–10. doi: 10.1182/blood-2011-02-336321, PMID: 21821703

[74] Mehta BA Schmidt-Wolf IG Weissman IL Negrin RS . Two pathways of exocytosis of cytoplasmic granule contents and target cell killing by cytokine-induced cd3+ Cd56+ Killer cells. Blood. (1995) 86:3493–9. doi: 10.1182/blood.V86.9.3493.bloodjournal8693493, PMID: 7579455

[75] Wu X Sharma A Oldenburg J Weiher H Essler M Skowasch D . Nkg2d engagement alone is sufficient to activate cytokine-induced killer cells while 2b4 only provides limited coactivation. Front Immunol. (2021) 12:731767. doi: 10.3389/fimmu.2021.731767, PMID: 34691037 PMC8529192

[76] Guo YL Han WD . Cytokine-induced killer (Cik) cells: from basic research to clinical translation. Chin J Cancer. (2015) 34:99–107. doi: 10.1186/s40880-015-0002-1, PMID: 25962508 PMC4593361

[77] Wongkajornsilp A Somchitprasert T Butraporn R Wamanuttajinda V Kasetsinsombat K Huabprasert S . Human cytokine-induced killer cells specifically infiltrated and retarded the growth of the inoculated human cholangiocarcinoma cells in scid mice. Cancer Invest. (2009) 27:140–8. doi: 10.1080/07357900802189832, PMID: 19235585

[78] Morisaki T Umebayashi M Kiyota A Koya N Tanaka H Onishi H . Combining cetuximab with killer lymphocytes synergistically inhibits human cholangiocarcinoma cells *in vitro*. Anticancer Res. (2012) 32:2249–56., PMID: 22641659

[79] Morisaki T Hirano T Koya N Kiyota A Tanaka H Umebayashi M . Nkg2d-directed cytokine-activated killer lymphocyte therapy combined with gemcitabine for patients with chemoresistant metastatic solid tumors. Anticancer Res. (2014) 34:4529–38., PMID: 25075096

[80] Wang J He M Shi WJ Sha HF Feng JX Wang SJ . Inducible costimulator (Icos) enhances the cytolytic activity of cytokine-induced killer cells against gallbladder cancer *in vitro* and *in vivo*. Cancer Invest. (2009) 27:244–50. doi: 10.1080/07357900802239124, PMID: 19194830

[81] He M Wang Y Shi WJ Wang SJ Sha HF Feng JX . Immunomodulation of Inducible Co-Stimulator (Icos) in Human Cytokine-Induced Killer Cells against Cholangiocarcinoma through Icos/Icos Ligand Interaction. J Dig Dis. (2011) 12:393–400. doi: 10.1111/j.1751-2980.2011.00527.x, PMID: 21955433

[82] Wu T Zhang LZ Zeng Z Yan T Cheng JM Miao XJ . Complete response to pd-1 inhibitor in primary hepatocellular carcinoma patients post-progression on bi-specific antibody conjugated cik cell treatment: A report of two cases. Onco Targets Ther. (2021) 14:5447–53. doi: 10.2147/ott.S333604, PMID: 34984004 PMC8702989

[83] Shao W Yao Y Yang L Li X Ge T Zheng Y . Novel insights into tcr-T cell therapy in solid neoplasms: optimizing adoptive immunotherapy. Exp Hematol Oncol. (2024) 13:37. doi: 10.1186/s40164-024-00504-8, PMID: 38570883 PMC10988985

[84] Ai HH Yang H Li L Ma J Liu KD Li Z . Cancer/testis antigens: promising immunotherapy targets for digestive tract cancers. Front Immunol. (2023) 14:1190883. doi: 10.3389/fimmu.2023.1190883, PMID: 37398650 PMC10311965

[85] Hong DS Van Tine BA Biswas S McAlpine C Johnson ML Olszanski AJ . Autologous T cell therapy for mage-A4(+) solid cancers in hla-a*02(+) patients: A phase 1 trial. Nat Med. (2023) 29:104–14. doi: 10.1038/s41591-022-02128-z, PMID: 36624315 PMC9873554

[86] Zhou JX Li Y Chen SX Deng AM . Expression and prognostic significance of cancer-testis antigens (Cta) in intrahepatic cholagiocarcinoma. J Exp Clin Cancer Res. (2011) 30:2. doi: 10.1186/1756-9966-30-2, PMID: 21211023 PMC3023685

[87] Chen XT Leisegang M Gavvovidis I Pollack SM Lorenz FKM Schumacher TN . Generation of effective and specific human tcrs against tumor/testis antigen ny-eso-1 in mice with humanized T cell recognition system. Front Immunol. (2024) 15:1524629. doi: 10.3389/fimmu.2024.1524629, PMID: 39776913 PMC11703889

[88] Kawai A Ishihara M Nakamura T Kitano S Iwata S Takada K . Safety and efficacy of ny-eso-1 antigen-specific T-cell receptor gene-transduced T lymphocytes in patients with synovial sarcoma: A phase I/ii clinical trial. Clin Cancer Res. (2023) 29:5069–78. doi: 10.1158/1078-0432.Ccr-23-1456, PMID: 37792433 PMC10722137

[89] Amissah OB Chen W de Dieu Habimana J Sun Y Lin L Liu Y . Ny-eso-1-specific T cell receptor-engineered T cells and tranilast, a trpv2 antagonist bivalent treatment enhances the killing of esophageal cancer: A dual-targeted cancer therapeutic route. Cancer Cell Int. (2024) 24:64. doi: 10.1186/s12935-024-03249-w, PMID: 38336680 PMC10858587

[90] Chinnasamy N Wargo JA Yu Z Rao M Frankel TL Riley JP . A tcr targeting the hla-a*0201-restricted epitope of mage-A3 recognizes multiple epitopes of the mage-a antigen superfamily in several types of cancer. J Immunol. (2011) 186:685–96. doi: 10.4049/jimmunol.1001775, PMID: 21149604 PMC6292200

[91] Ishii T Yasuchika K Suemori H Nakatsuji N Ikai I Uemoto S . Alpha-fetoprotein producing cells act as cancer progenitor cells in human cholangiocarcinoma. Cancer Lett. (2010) 294:25–34. doi: 10.1016/j.canlet.2010.01.019, PMID: 20149523

[92] Zhu W Zhang ZM Chen JZ Chen XL Huang L Zhang XY . A novel engineered il-21 receptor arms T-cell receptor-engineered T cells (Tcr-T cells) against hepatocellular carcinoma. Signal Transduct Target Ther. (2024) 9:101. doi: 10.1038/s41392-024-01792-6, PMID: 38643203 PMC11032311

[93] Meyer T Finn RS Borad M Mahipal A Edeline J Houot R . Phase I trial of adp-A2afp tcr T-cell therapy in patients with advanced hepatocellular or gastric hepatoid carcinoma. J Hepatol. (2026) 84(1):113–21. doi: 10.1016/j.jhep.2025.07.033, PMID: 40812667

[94] Cai L Galva LDC Peng YB Luo XB Zhu W Yao YH . Preclinical studies of the off-target reactivity of afp158-specific tcr engineered T cells. Front Immunol. (2020) 11:607. doi: 10.3389/fimmu.2020.00607, PMID: 32395117 PMC7196607

[95] Nakamura H Arai Y Totoki Y Shirota T Elzawahry A Kato M . Genomic spectra of biliary tract cancer. Nat Genet. (2015) 47:1003–10. doi: 10.1038/ng.3375, PMID: 26258846

[96] Morelli MP Negrao MV Collinson-Pautz MR Jernigan K Demars NA Weitzman A . Safety and efficacy of sleeping beauty tcr-T cells targeting shared kras and tp53 mutations expressed by solid tumors in first-in-human phase 1 study. J Clin Oncol. (2023) 41:2547. doi: 10.1200/JCO.2023.41.16_suppl.2547

[97] Hanahan D Michielin O Pittet MJ . Convergent inducers and effectors of T cell paralysis in the tumour microenvironment. Nat Rev Cancer. (2025) 25:41–58. doi: 10.1038/s41568-024-00761-z, PMID: 39448877

[98] Arner EN Rathmell JC . Metabolic programming and immune suppression in the tumor microenvironment. Cancer Cell. (2023) 41:421–33. doi: 10.1016/j.ccell.2023.01.009, PMID: 36801000 PMC10023409

[99] Chen S Li S Wang H . Remodeling tumor-associated macrophages in the tumor microenvironment. Oncol Trans Med. (2024) 10:281–5. doi: 10.1097/ot9.0000000000000063

[100] Moon EK Carpenito C Sun J Wang LCS Kapoor V Predina J . Expression of a functional ccr2 receptor enhances tumor localization and tumor eradication by retargeted human T cells expressing a mesothelin-specific chimeric antibody receptor. Clin Cancer Res. (2011) 17:4719–30. doi: 10.1158/1078-0432.Ccr-11-0351, PMID: 21610146 PMC3612507

[101] Jafarzadeh L Masoumi E Fallah-Mehrjardi K Mirzaei HR Hadjati J . Prolonged persistence of chimeric antigen receptor (Car) T cell in adoptive cancer immunotherapy: challenges and ways forward. Front Immunol. (2020) 11:702. doi: 10.3389/fimmu.2020.00702, PMID: 32391013 PMC7188834

[102] Tseng D Lee S . Tumor-infiltrating lymphocyte therapy: A new frontier. Transplant Cell Ther. (2025) 31:S599–609. doi: 10.1016/j.jtct.2024.11.014, PMID: 40089329

[103] Liu SC Meng Y Liu L Lv YG Yu WW Liu T . Cd4+ T cells are required to improve the efficacy of cik therapy in non-small cell lung cancer. Cell Death Dis. (2022) 13:441. doi: 10.1038/s41419-022-04882-x, PMID: 35523765 PMC9076680

[104] van Ostaijen-ten Dam MM Prins HJ Boerman GH Vervat C Pende D Putter H . Preparation of cytokine-activated nk cells for use in adoptive cell therapy in cancer patients: protocol optimization and therapeutic potential. J Immunother. (2016) 39:90–100. doi: 10.1097/cji.0000000000000110, PMID: 26849078

[105] Zhai Y Du Y Li G Yu M Hu H Pan C . Trogocytosis of car molecule regulates car-T cell dysfunction and tumor antigen escape. Signal Transduct Target Ther. (2023) 8:457. doi: 10.1038/s41392-023-01708-w, PMID: 38143263 PMC10749292

[106] Lin H Yang X Ye S Huang L Mu W . Antigen escape in car-T cell therapy: mechanisms and overcoming strategies. BioMed Pharmacother. (2024) 178:117252. doi: 10.1016/j.biopha.2024.117252, PMID: 39098176

[107] Vijay V Karisani N Shi L Hung YH Vu P Kattel P . Generation of a biliary tract cancer cell line atlas identifies molecular subtypes and therapeutic targets. Cancer Discov. (2025) 15:1858–82. doi: 10.1158/2159-8290.Cd-24-1383, PMID: 40353839 PMC12353624

[108] Giraldo NA Drill E Satravada BA El Dika I Brannon AR Dermawan J . Comprehensive molecular characterization of gallbladder carcinoma and potential targets for intervention. Clin Cancer Res. (2022) 28:5359–67. doi: 10.1158/1078-0432.Ccr-22-1954, PMID: 36228155 PMC9772093

[109] Silver AB Wang J Spangler JB . Masking T cell engagers mitigates on-target off-tumor activity. Nat Cancer. (2023) 4:439–41. doi: 10.1038/s43018-023-00529-8, PMID: 36997746

[110] Mao ZY Zhu GQ Ren L Guo XC Su D Bai L . Prognostic value of C-met expression in cholangiocarcinoma. Technol Cancer Res Treat. (2016) 15:227–33. doi: 10.1177/1533034615578959, PMID: 25873560

[111] Wei ZY Xu JY Zhao CK Zhang M Xu N Kang LQ . Prediction of severe crs and determination of biomarkers in B cell-acute lymphoblastic leukemia treated with car-T cells. Front Immunol. (2023) 14:1273507. doi: 10.3389/fimmu.2023.1273507, PMID: 37854590 PMC10579557

[112] Schubert ML Schmitt M Wang L Ramos CA Jordan K Müller-Tidow C . Side-effect management of chimeric antigen receptor (Car) T-cell therapy. Ann Oncol. (2021) 32:34–48. doi: 10.1016/j.annonc.2020.10.478, PMID: 33098993

[113] Liu Y Zhou N Zhou L Wang J Zhou Y Zhang T . Il-2 regulates tumor-reactive cd8(+) T cell exhaustion by activating the aryl hydrocarbon receptor. Nat Immunol. (2021) 22:358–69. doi: 10.1038/s41590-020-00850-9, PMID: 33432230

[114] Tartour E Mathiot C Fridman WH . Current status of interleukin-2 therapy in cancer. BioMed Pharmacother. (1992) 46:473–84. doi: 10.1016/0753-3322(92)90005-r, PMID: 1306361

[115] Malek TR . The main function of il-2 is to promote the development of T regulatory cells. J Leukoc Biol. (2003) 74:961–5. doi: 10.1189/jlb.0603272, PMID: 12960253

[116] Zorn E Nelson EA Mohseni M Porcheray F Kim H Litsa D . Il-2 regulates foxp3 expression in human cd4+Cd25+ Regulatory T cells through a stat-dependent mechanism and induces the expansion of these cells *in vivo*. Blood. (2006) 108:1571–9. doi: 10.1182/blood-2006-02-004747, PMID: 16645171 PMC1895505

[117] Hammer MH Meyer S Brestrich G Moosmann A Kern F Tesfa L . Hla type-independent generation of antigen-specific T cells for adoptive immunotherapy. Eur J Immunol. (2005) 35:2250–8. doi: 10.1002/eji.200526230, PMID: 15915543

[118] Wang X Song X Li Y Ding Y Yin C Ren T . Integrated system for screening tumor-specific tcrs, epitopes, and hla subtypes using single-cell sequencing data. J Immunother Cancer. (2025) 13:e012029. doi: 10.1136/jitc-2025-012029, PMID: 40744664 PMC12314965

[119] Qiao Y Chen J Wang X Yan S Tan J Xia B . Enhancement of car-T cell activity against cholangiocarcinoma by simultaneous knockdown of six inhibitory membrane proteins. Cancer Commun (Lond). (2023) 43:788–807. doi: 10.1002/cac2.12452, PMID: 37282786 PMC10354409

[120] Chen AXY Yap KM Kim JS Sek K Huang YK Dunbar PA . Rewiring endogenous genes in car T cells for tumour-restricted payload delivery. Nature. (2025) 644:241–51. doi: 10.1038/s41586-025-09212-7, PMID: 40604285 PMC12328239

[121] Kunert A Chmielewski M Wijers R Berrevoets C Abken H Debets R . Intra-tumoral production of il18, but not il12, by tcr-engineered T cells is non-toxic and counteracts immune evasion of solid tumors. Oncoimmunology. (2017) 7:e1378842. doi: 10.1080/2162402x.2017.1378842, PMID: 29296541 PMC5739571

[122] Escobar G Berger TR Maus MV . Car-T cells in solid tumors: challenges and breakthroughs. Cell Rep Med. (2025) 6:102353. doi: 10.1016/j.xcrm.2025.102353, PMID: 40945517 PMC12711667

[123] Wu CY Roybal KT Puchner EM Onuffer J Lim WA . Remote control of therapeutic T cells through a small molecule-gated chimeric receptor. Science. (2015) 350:aab4077. doi: 10.1126/science.aab4077, PMID: 26405231 PMC4721629

[124] Giordano Attianese GMP Shui S Cribioli E Triboulet M Scheller L Hafezi M . Dual on/off-switch chimeric antigen receptor controlled by two clinically approved drugs. Proc Natl Acad Sci U.S.A. (2024) 121:e2405085121. doi: 10.1073/pnas.2405085121, PMID: 39453747 PMC11536088

[125] Timpanaro A Piccand C Dzhumashev D Anton-Joseph S Robbi A Moser J . Cd276-car T cells and dual-car T cells targeting cd276/fgfr4 promote rhabdomyosarcoma clearance in orthotopic mouse models. J Exp Clin Cancer Res. (2023) 42:493. doi: 10.1186/s13046-023-02838-3, PMID: 37924157 PMC10625270

[126] Qin H Zhou Z Shi R Mai Y Xu Y Peng F . Insights into next-generation immunotherapy designs and tools: molecular mechanisms and therapeutic prospects. J Hematol Oncol. (2025) 18:62. doi: 10.1186/s13045-025-01701-6, PMID: 40483473 PMC12145627

[127] Guo J Wang C Luo N Wu Y Huang W Zhu J . Il-2-free tumor-infiltrating lymphocyte therapy with pd-1 blockade demonstrates potent efficacy in advanced gynecologic cancer. BMC Med. (2024) 22:207. doi: 10.1186/s12916-024-03420-0, PMID: 38769543 PMC11106999

[128] Chong EA Alanio C Svoboda J Nasta SD Landsburg DJ Lacey SF . Pembrolizumab for B-cell lymphomas relapsing after or refractory to cd19-directed car T-cell therapy. Blood. (2022) 139:1026–38. doi: 10.1182/blood.2021012634, PMID: 34496014 PMC9211527

[129] Liu SC Meng Y Liu L Lv YG Wei F Yu WW . Rational pemetrexed combined with cik therapy plus anti-pd-1 mabs administration sequence will effectively promote the efficacy of cik therapy in non-small cell lung cancer. Cancer Gene Ther. (2023) 30:277–87. doi: 10.1038/s41417-022-00543-5, PMID: 36352092

[130] Du J Su S Li HY Shao J Meng FY Yang M . Low dose irradiation increases adoptive cytotoxic T lymphocyte migration in gastric cancer. Exp Ther Med. (2017) 14:5711–6. doi: 10.3892/etm.2017.5305, PMID: 29285113 PMC5740708

[131] Klug F Prakash H Huber PE Seibel T Bender N Halama N . Low-dose irradiation programs macrophage differentiation to an inos+/M1 phenotype that orchestrates effective T cell immunotherapy. Cancer Cell. (2013) 24:589–602. doi: 10.1016/j.ccr.2013.09.014, PMID: 24209604

[132] Kumar A Fischer C Cichocki F Miller JS . Multiplexed ipsc platform for advanced nk cell immunotherapies. Cell Rep Med. (2025) 6:102282. doi: 10.1016/j.xcrm.2025.102282, PMID: 40780202 PMC12711691

[133] He Q Liu Z Liu Z Lai Y Zhou X Weng J . Tcr-like antibodies in cancer immunotherapy. J Hematol Oncol. (2019) 12:99. doi: 10.1186/s13045-019-0788-4, PMID: 31521180 PMC6744646

[134] Marrer-Berger E Nicastri A Augustin A Kramar V Liao H Hanisch LJ . The physiological interactome of tcr-like antibody therapeutics in human tissues. Nat Commun. (2024) 15:3271. doi: 10.1038/s41467-024-47062-5, PMID: 38627373 PMC11021511

[135] Biber G Sabag B Raiff A Ben-Shmuel A Puthenveetil A Benichou JIC . Modulation of intrinsic inhibitory checkpoints using nano-carriers to unleash nk cell activity. EMBO Mol Med. (2022) 14:e14073. doi: 10.15252/emmm.202114073, PMID: 34725941 PMC8749471

[136] Li X Halldórsdóttir HR Weller S Colliander A Bak M Kempen P . Enhancing adoptive cell therapy by T cell loading of shp2 inhibitor nanocrystals before infusion. ACS Nano. (2022) 16:10918–30. doi: 10.1021/acsnano.2c03311, PMID: 35838499

[137] Weiss L Weiden J Dölen Y Grad EM van Dinther EAW Schluck M . Direct *in vivo* activation of T cells with nanosized immunofilaments inhibits tumor growth and metastasis. ACS Nano. (2023) 17:12101–17. doi: 10.1021/acsnano.2c11884, PMID: 37338806 PMC10339791

[138] Parayath NN Stephan SB Koehne AL Nelson PS Stephan MT . *In vitro*-transcribed antigen receptor mrna nanocarriers for transient expression in circulating T cells *in vivo*. Nat Commun. (2020) 11:6080. doi: 10.1038/s41467-020-19486-2, PMID: 33247092 PMC7695830

[139] Chen Q Sun J Ling S Yang H Li T Yang X . Tumor microenvironment-responsive nano-immunomodulators for enhancing chimeric antigen receptor-T cell therapy in lung cancer. ACS Nano. (2025) 19:8212–26. doi: 10.1021/acsnano.4c17899, PMID: 39988897

[140] Gentilini A Pastore M Marra F Raggi C . The role of stroma in cholangiocarcinoma: the intriguing interplay between fibroblastic component, immune cell subsets and tumor epithelium. Int J Mol Sci. (2018) 19(10):2885. doi: 10.3390/ijms19102885, PMID: 30249019 PMC6213545

[141] Lin Y Li B Yang X Cai Q Liu W Tian M . Fibroblastic fap promotes intrahepatic cholangiocarcinoma growth via mdscs recruitment. Neoplasia. (2019) 21:1133–42. doi: 10.1016/j.neo.2019.10.005, PMID: 31759251 PMC6880109

[142] Das S Valton J Duchateau P Poirot L . Stromal depletion by talen-edited universal hypoimmunogenic fap-car T cells enables infiltration and anti-tumor cytotoxicity of tumor antigen-targeted car-T immunotherapy. Front Immunol. (2023) 14:1172681. doi: 10.3389/fimmu.2023.1172681, PMID: 37251405 PMC10213512

[143] Shahvali S Rahiman N Jaafari MR Arabi L . Targeting fibroblast activation protein (Fap): advances in car-T cell, antibody, and vaccine in cancer immunotherapy. Drug Delivery Transl Res. (2023) 13:2041–56. doi: 10.1007/s13346-023-01308-9, PMID: 36840906

[144] Hua S Gu X Jin H Zhang X Liu Q Yang J . Tumor-infiltrating T lymphocytes: A promising immunotherapeutic target for preventing immune escape in cholangiocarcinoma. BioMed Pharmacother. (2024) 177:117080. doi: 10.1016/j.biopha.2024.117080, PMID: 38972151

[145] Zhang L Tian L Dai X Yu H Wang J Lei A . Pluripotent stem cell-derived car-macrophage cells with antigen-dependent anti-cancer cell functions. J Hematol Oncol. (2020) 13:153. doi: 10.1186/s13045-020-00983-2, PMID: 33176869 PMC7656711

[146] Maalej KM Merhi M Inchakalody VP Mestiri S Alam M Maccalli C . Car-cell therapy in the era of solid tumor treatment: current challenges and emerging therapeutic advances. Mol Cancer. (2023) 22:20. doi: 10.1186/s12943-023-01723-z, PMID: 36717905 PMC9885707

[147] Cao Q Wang Y Chen J Wang R Chen T Gloss B . Targeting inflammation with chimeric antigen receptor macrophages using a signal switch. Nat BioMed Eng. (2025) 9:1502–16. doi: 10.1038/s41551-025-01387-8, PMID: 40335685 PMC12443588

[148] Xiao Z Wang J He S Wang L Yang J Li W . Engineered T cells stimulate dendritic cell recruitment and antigen spreading for potent anti-tumor immunity. Cell Rep Med. (2025) 6:102307. doi: 10.1016/j.xcrm.2025.102307, PMID: 40858102 PMC12490219

